# Natural occurrence of infections of the spittlebug *Philaenus spumarius* by the entomopathogenic fungi *Zoophthora radicans* and *Batkoa major* in Northwest Italy

**DOI:** 10.3897/mycokeys.128.181516

**Published:** 2026-02-18

**Authors:** Paola Dolci, Enrique Quesada-Moraga, Cezary Tkaczuk, Francesco Volpe, Simona Abbà, Alessandro Cicerone, Marika Rossi, Marta Vallino, Domenico Bosco

**Affiliations:** 1 Department of Agricultural, Forest and Food Sciences, University of Turin, Largo Braccini 2, Grugliasco, Italy Institute for Sustainable Plant Protection-CNR Turin Italy; 2 Department of Agronomy, ETSIAM, University of Cordoba, Campus de Rabanales, Ctra. Madrid km 396, Cordoba, Spain Department of Horticulture and Plant Protection, University of Siedlce Siedlce Poland; 3 Department of Horticulture and Plant Protection, University of Siedlce, Bolesława Prusa 14, Siedlce, Poland Forest and Food Sciences, University of Turin Grugliasco Italy; 4 Institute for Sustainable Plant Protection-CNR, Strada delle Cacce 73, Turin, Italy ETSIAM, University of Cordoba Cordoba Spain

**Keywords:** Entomophthorales, natural infection, pest

## Abstract

An infection event of the spittlebug *Philaenus spumarius* (Hemiptera, Aphrophoridae) has been described, for the first time, in northwest Italy. The causative agents were two entomopathogenic fungi belonging to Entomophthorales, specifically *Zoophthora radicans* and, more rarely, *Batkoa major*. The morphological description and molecular identification of fungi have been reported, in addition to recording meteorological data that may have affected the outbreak of the infection. When massive events are ongoing, entomopathogenic fungi really behave as determinant regulators of natural populations of arthropod pests and the possibility to stress their action in this direction should be deeply investigated.

## Introduction

The spittlebug *Philaenus spumarius* (Hemiptera, Aphrophoridae), a ubiquitous and polyphagous insect, is the main vector in Europe of the bacterium *Xylella fastidiosa*, namely of the subsp. *pauca* sequence type (ST53), the causal agent of the olive quick decline syndrome (OQDS) that led recently to a dramatic dieback of olive trees in Apulia (South Italy), and of the subsp. fastidiosa (ST1), causing Pierce’s Disease of grapevine. In Italy, *P. spumarius* is present in all regions and, although *X. fastidiosa* has been found only in a few regions (Apulia, Tuscany and Lazio), it is regarded as a potential vector subjected to monitoring campaigns.

In Apulia, current control strategies targeting vector populations primarily rely on agronomic measures such as soil tilling that interfere, in spring, with nymph development, reducing spittlebug populations. Differently, the impact of insecticidal application on olive trees, against the adult stage, remains unclear with controversial results, being the few active ingredients available characterised by low persistence.

Despite *P. spumarius* being considered a major phytosanitary issue, there is a significant lack of information about its natural biological control agents (Cornara et al. 2018). To date, few works report the importance of entomopathogenic fungi (EPF) belonging to order Hypocreales as promising candidates for biological control of the spittlebug and data are available on *Metarhizium brunneum* and *Beauveria bassiana* (Yousef-Yousef et al. 2023), *Lecanicillium aphanocladii* (Bodino et al. 2024), *Trichoderma* sp. (Ganassi et al. 2023). The present study describes, for the first time, a natural major infection event of *P. spumarius* affected by Entomophthorales, in northwest Italy. Actually, in 2018, in a nearby area, our group described a single *P. spumarius* individual naturally affected by *Conidiobolus coronatus* as a one-time event (Bodino et al. 2024). Entomophthoralean species have been found naturally infecting and causing epizootics in different pests worldwide. For instance, infections of spittlebugs by *Batkoa* and *Furia* in Brazil (Leite et al. 2002) and *Pandora* in Argentina were described in pastures (Foieri et al. 2017). Similarly, cicadelids were found succumbing to infections caused by Entomophthorales, and the occurrence of *Zoophthora* sp. in populations of leafhoppers has been reported in several countries (Ben-Ze’ev and Kenneth 1981; Galaini-Wraight et al. 1991; Mazzoglio et al. 2009). In South America, the genera *Pandora* and *Conidiobolus* were recorded occurring on adults of planthoppers associated with rice crops (Toledo et al. 2008). Weaver and King (1954) reported the attack of *P. spumarius* by *Entomophthora* sp. in Denmark.

In our study, a detailed morphological and molecular description of the two entomophthoralean fungi causing *P. spumarius* infection is provided.

## Materials and methods

*P. spumarius* individuals affected by mycosis were found dead attached to the adaxial and abaxial leaf surfaces of herbaceous cover of a vineyard located in Piedmont (44°55.285'N, 8°11.78'E). Two surveys for the collection of cadavers were carried out on 14^th^ and 28^th^ May 2024 and the average temperature, relative humidity and precipitation were recorded daily in April and May 2024 by a weather station located 1.2 km away from the vineyard. The last inspection was carried out on June 10 when no more individuals affected by mycosis were found. Fungal incidence was calculated as the average number of cadavers per leaf showing sign of infection. Dead insects attached to leaves were placed, individually, in Petri dishes and brought to the laboratory. Cadavers were first observed and photographed using a binocular stereomicroscope equipped with a Leica EC4 camera and imaging software for automatic measurements (LAS-EZ, Leica Microsystems Application Suite, Switzerland). Fungal material directly taken from the cadavers was, then, carefully dissected and mounted on lactic acid slides to observe and measure morphological structures by optical microscope. The production of primary conidia from insects was stimulated according to ‘descending conidia’ showering method (Hajek et al. 2012). Briefly, *P. spumarius* cadavers were attached to the underside of the lid of a Petri dish on a moistened piece of sterile filter paper. The lid was then placed over the Petri dish base containing a slide, and incubated at 23 °C. Primary conidia discharged from the cadavers were observed and measured after overnight incubation and nuclei stained directly on the microscope slide by incubation for 1 min with few drops of a 1 ug/mL DAPI/water solution. Fluorescent nuclei were observed, and images acquired, with a Leica DM750 microscope equipped with a CoolLED pE300white Illumination System, using the LED cube filter at 365 nm. All measurements were based, if not otherwise stated, on n = 25 structures.

In order to support morphological classification, molecular identification was also performed. DNA was extracted from dead insects using the CTAB method (Marzachì et al. 1998), and the nuclear small subunit of ribosomal DNA sequence was PCR-amplified by primers nu-SSU-00021-5’(Gargas and DePriest 1996) and nu-SSU-1780-3’ (DePriest 1993). DNA amplicons were cloned into pGEM-T plasmid (Promega, Milan, Italy) and sequenced using M13 universal primers.

## Results

*P. spumarius* adults and nymphs were found affected by mycosis at an epizootic level. Occasionally, 4^th^-5^th^ instar nymphs were also observed. On average, 3 cadavers/leaf were found on *Dittrichia viscosa* (L.) Greuter, a preferred species for *P. spumarius* (Mesmin et al., 2022; author’s observations). Data of temperature, rainfall and relative humidity were recorded during the infection event and in the month before. Daily average temperature was 12.55 °C (T_min_ -0.40 °C; T_max_ 26.70 °C) and 16.16 °C (T_min_ 6.70 °C; T_max_ 25.50 °C), in April and May 2024, respectively. Rainfall reached a total of 126.70 mm in April while the value fell to 58.00 mm in May. The average relative humidity was 75.3% (RH_min_ 15.0%; RH_max_ 99.0%) in April and 81.4% (RH_min_ 38.0%; RH_max_ 99.0%) in May.

**Figure 1.**
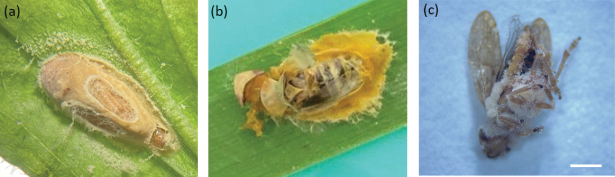
*Philaenus spumarius* cadavers covered by mycelium attributable to the morphotype E1 and surrounded by a creamy white halo of projected conidia (**a**) or characterized by elongated abdomen (**b**); *P. spumarius* cadaver covered by mycelium attributable to the morphotype E2 (**c**). Scale bar: 1.3 mm

**Figure 2.**
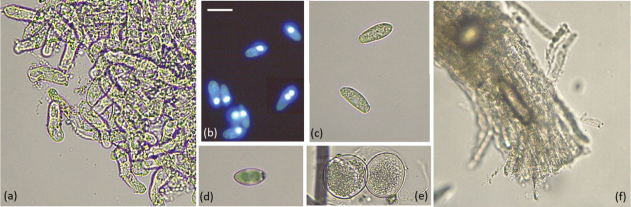
Morphological structures of morphotype E1 attributable to the species *Zoophthora radicans*. Hyphal segments (**a**); uninucleate primary conidia, rarely binucleate (**b, c**); secondary bitunicate conidia (**d**); hialine resting spores (**e**); rhizoid (**f**). Scale bars: 22 µm (**a**); 20 µm (**b, c, d, e**).

In total, 98 insects affected by mycosis were randomly collected. Two different fungal morphotypes, E1 (Fig. 1a, b) and E2 (Fig. 1c), were differentiated at stereomicroscope: the yellowish and sticky mycelium of 95 insects was attributable to the morphotype E1, the whitish mycelium of 3 insects to the morphotype E2. Some cadavers belonging to morphotype E1 showed swollen elongated abdomens (Fig. 1b) and primary conidia discharged on leaf surface around the body (Fig. 1a). The morphological structures were described and measured for the fungi grown on and taken directly from cadavers, except for conidia obtained by ‘descending conidia’ method. The mycelium filling the host hemocoel consisted of hyphal segments with a diameter of 8.9 (6.3–11.6) ± 1.8 µm (Fig. 2a). Primary conidia were elongate ellipsoid (Fig. 2b, c), 20.47 (15.6–24.5) ± 1.9 µm long and 9.3.

(6.7–11.6) ± 1.2 µm wide, uninucleate, rarely binucleate (Fig. 2b); secondary conidia (n = 3) were bitunicate with a thin outer layer and a conical papilla demarcated with a slight protuberance (Fig. 2d). Unfortunately, no capillospores were detected. Resting spores were hyaline with 28.5 ± 3.0 µm diameter (n = 2, Fig. 2e). Cadavers were found attached to leaves by thin rhizoids (n = 3, Fig. 2f) particularly abundant in thoracic part. Morphological characteristics of morphotype E1 matched those reported by Bałazy (1993) for the species *Zoophthora radicans* (Brefeld) Batko (Zygomycota, Entomophthorales).

**Figure 3.**
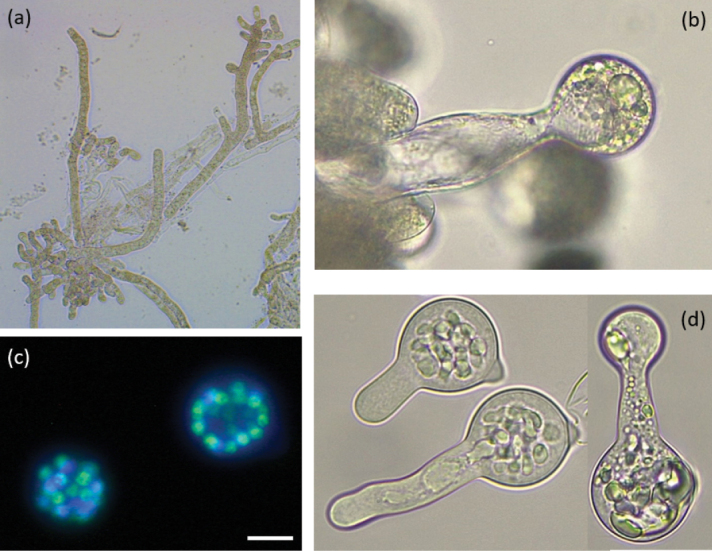
Morphological structure of morphotype E2 attributable to the species *Batkoa major*. Branching hyphae (**a**); conidiophore with narrow neck between conidiogenous cell and conidium (**b**); multinucleate primary conidia (**c**); primary and secondary conidia (**d**). Scale bars: 65 µm (**a**); 16 µm (**b, c, d**).

Hyaline and branching hyphae with 13.6 (11.6–18.5) ± 2.9 µm diameter were observed for morphotype E2 (Fig. 3a). Unbranched conidiophores, bearing a single apical primary conidium, measured 20.1 (18.1–22.0) ± 0.9 µm diameter (n = 12) (Fig. 3b). Primary globose multinucleate conidia, containing 18–25 nuclei, of (40.0) 44.7 (47.2) µm length and (38.6) 37.5 (34.8) µm width, with prominent conical papilla, and forming smaller secondary conidia are reported in Fig. 3c, d. Morphological characteristics of morphotype E2 matched those reported by Bałazy (1993) for the species *Batkoa major* (Thaxt.) Humber.

The NCBI BLASTn search against the non-redundant nucelotide (nt) database showed 96.2% identity of morphotype E1 with *Z. radicans* (GenBank accession: MG253005.1), and 99.5% identity of morphotype E2 with *Batkoa major* (GenBank accession: EF392559.1) confirming the morphological identification. Sequences of DNA amplicons were deposited to NCBI GenBank database under the accession numbers: PX415466 (E1) and PX415467 (E2).

## Discussion

This study provides the first record of a massive infection event of *P. spumarius* by the EPF*Z. radicans* and, with a secondary association involving *Batkoa major*. It is known that the natural incidence of EPF in insect populations is influenced by environmental factors. An average daily relative humidity (RH) below 80% is unfavourable for many Entomophthorales fungi (Oduor et al. 1996). On the other hand, heavy rainfall can lower the infection rates by washing conidia off dead insects, thereby reducing the inoculum density (Leite et al. 2002; Foieri et al. 2017). In our data, the average RH in May 2024 was 81.4%, which is 6.1 percentage points higher than in April (75.3%). Additionally, total rainfall decreased from 126.70 mm in April to 58.00 mm in May. These environmental conditions likely contributed to the outbreak observed in May.

The individuals affected by mycosis were mainly adults but a few 4^th^–5^th^ instar nymphs were also found which can be explained both by the timing of *P. spumarius* life cycle and by the protection of spittle mass produced by nymphs that creates a suitable microclimate, as also observed by Foieri et al. (2017).

Fungal species within the order of Entomophthorales are known to be associated with the Hemiptera. They show rapid sporulation and host invasion, and an efficient dispersal from forcible conidia discharge which make them often associated with epizootics in their host population (Torres Acosta et al. 2016). *Z. radicans* is a typical representative of this order and can attack members of Aphidae, Cercopidae, Cicadellidae, Delphacidae, Miridae, Pentatomidae, Psyllidae and Triozidae families (Humber 1992; Hajek et al. 2025), besides insects belonging to the orders of Coleoptera, Diptera, Hymenoptera and Lepidoptera (Keller 1991; Humber 1992). Over the years, a natural occurrence of *Z. radicans* was reported mainly in USA (Hajek et al. 2025) and South America (Mascarin et al. 2012; Torres Acosta et al. 2016; Manfrino et al. 2018) besides Australia (Batta 2011), China (Huang 2008) and Zimbabwe (Manyangarirwa 2011). In Italy, *Z. radicans* was found in 1986 as one of the natural enemies of leafhoppers (Ozino and Zeppa 1988) and, more recently, it was recognized to be the most effective pathogen of *Zyginidia pullula* (Boheman) (Mazzoglio et al. 2009). In the present work, morphological identification of *Z. radicans* was primarily reached by recording shape, size and number of nuclei in conidia (Balazy 1993), in addition to the observation of representative structures such as adhesive rhyzoids produced by the fungus to block the insect on the leaves, and resistant spores for long term survival. The analysis and blasting in GenBank of the nuclear small subunit (SSU) rRNA gene sequence confirmed morphological identification for *Z. radicans*.

*B. major* has a broad host range, infecting insect species across 5 orders and, among Hemiptera, causes mycosis in members of Aphidae, Cicadellidae, Delphacidae and Fulgoridae families (Gryganskyi et al. 2022; Hajek et al. 2023). This fungus is a widespread species worldwide and, in Europe, has been detected in Sweden, Poland, Scotland, Switzerland and Austria (Hajek et al. 2023). Our morphological observations from the cadavers of host insects do not differ from previously published records (Thaxter 1888; Balazy 1993) even if primary conidia diameters are a bit lower. Considerable variability in the morphology of *B. major* has been reported by other authors (Hajek et al. 2023) and the 99.5% genetic similarity proves rather indisputably that morphotype E2 belongs to *B. major*.

Although quantitative data is limited, evidence shows that insect populations often harbour diverse EPF, though outbreaks rarely reach epidemic levels (Gielen et al. 2024). During major events, EPF can act as key regulators of pest populations, a role that deserves deeper investigation.

Future research will monitor *P. spumarius* infections by *Z. radicans* and *B. major* in Piedmont to assess how environmental conditions influence their recurrence and improve our understanding of the ecological dynamics of these fungi.

## Additional information

### Use of AI

No use of AI was reported.

### Author ORCIDs

Paola Dolci https://orcid.org/0000-0003-1847-2367

Enrique Quesada-Moraga https://orcid.org/0000-0003-4021-3900

Cezary Tkaczuk https://orcid.org/0000-0002-4096-2154

Simona Abbà https://orcid.org/0000-0001-9345-5407

Marika Rossi https://orcid.org/0000-0001-8475-7366

Marta Vallino https://orcid.org/0000-0003-1465-3574

Domenico Bosco https://orcid.org/0000-0003-3945-2752

### Data availability

All of the data that support the findings of this study are available in the main text.
